# Sex and the city: Differences in disease- and disability-free life years, and active community participation of elderly men and women in 7 cities in Latin America and the Caribbean

**DOI:** 10.1186/1471-2458-8-127

**Published:** 2008-04-21

**Authors:** Angela MC Rose, Anselm J Hennis, Ian R Hambleton

**Affiliations:** 1 Chronic Disease Research Centre, Tropical Medicine Research Institute, The University of the West Indies, Barbados; 2 School of Clinical Medicine and Research, The University of the West Indies, Barbados

## Abstract

**Background:**

The world's population is ageing, and four of the top 10 most rapidly ageing developing nations are from the region of Latin America and the Caribbean (LAC).

Although an ageing population heralds likely increases in chronic disease, disability-related dependence, and economic burden, the societal contribution of the chronically ill or those with disability is not often measured.

**Methods:**

We calculated country-specific prevalences of 'disability' (difficulty with at least one activity of daily living), 'disease' and 'co-morbidity' (presence of at least one, and at least two, of seven chronic diseases/conditions, respectively), and 'active community engagement' (using five levels of community participation, from less than weekly community contact to voluntary or paid work) in seven LAC cities. We estimated remaining life expectancy (LE) with and without disability, disease and co-morbidity, and investigated age, sex, and regional variations in disability-free LE. Finally, we modeled the association of disease, co-morbidity and disability with active community participation using an ordinal regression model, adjusted for depression.

**Results:**

Overall, 77% of the LAC elderly had at least one chronic disease/condition, 44% had co-morbidity and 19% had a disability. The proportion of disability-free LE declined between the youngest (60–64 years) and the eldest (90 years and over) age-groups for both men (from 85% to 55%) and women (from 75% to 45%). Disease-free and co-morbidity-free LE, however, remained at approximately 30% and 62%, respectively, for men (20% and 48% for women), until 80–84 years of age, then increased. Only Bridgetown's participants had statistically significantly longer disability-free LE than the regional average (IRR = 1.08; 95%CI 1.05–1.10; p < 0.001). Only Santiago's participants had disability-free LE which was shorter than the regional average (IRR = 0.94; 95%CI 0.92–0.97; p < 0.001). There was 75% active community participation overall, with more women than men involved in active help (49% vs 32%, respectively) and more men involved in voluntary/paid work (46% vs 25%, respectively). There was either no, or borderline significance in the association between having one or more diseases/conditions and active community engagement for both sexes. These associations were limited by depression (odds ratio [OR] reduced by 15–17% for men, and by 8–11% for women), and only remained statistically significant in men. However, disability remained statistically significantly associated with less community engagement after adjusting for depression (OR = 0.58, 95%CI 0.49–0.69, p < 0.001 for women and OR = 0.50, 95%CI 0.47–0.65, p < 0.001 for men).

**Conclusion:**

There is an increasing burden of disease and disability with older age across the LAC region. As these nations cope with resulting social and economic demands, governments and civic societies must continue to develop and maintain opportunities for community participation by this increasingly frail, but actively engaged group.

## Background

The ageing of the world's population is now well documented, and projections indicate that this trend will continue throughout the first half of the current century [1]. The region of Latin America and the Caribbean (LAC) is experiencing rapid population ageing: four countries feature in the 10 most rapidly ageing developing nations [1]. Between 2004 and 2050, the population of this region is expected to increase by 42%, while the number of adults over 65 years will quadruple to about 136 million, an increase from about 4% to 17% of the population [2].

Initially discussed 20 years ago [3], Rowe and Kahn's definition of 'successful ageing' incorporates three elements: lack of disease and disability; remaining productively engaged in social activities; and maintaining high physical and cognitive function [4]. Researchers have continued to redefine this concept, and a recent review found 28 separate studies containing 29 different definitions [5]. Most definitions included self-perception of good health, as well as lack of disability; the authors concluded that this was probably due to the popularity of the Rowe and Kahn model. Other studies have shown that self-rated successful ageing does not correlate with absence of chronic disease and physical disability [6], and some have modified the model's first aspect to consider a more inclusive (and perhaps more realistic) concept of 'minimal' rather than 'no' disease [7].

We believe that labeling some of the elderly community as having 'successfully' aged implies that the others have somehow 'failed', and prefer the phrase 'active ageing'. This was defined by the World Health Organization (WHO) as "the process of optimizing opportunities for health, participation and security in order to enhance quality of life as people age [8]." Whatever the terminology, these definitions take us beyond the notion that lack of disease is the only goal of the health of the elderly, and lead us to consider other factors that may affect quality of life, such as the ability to actively participate in the community.

Taking the Rowe and Kahn model and the WHO definition as a framework, we investigate two aspects of active ageing in the LAC region using data from seven urban centres: levels of (1) disease and disability; and (2) active engagement in the community.

## Methods

The Survey on Health, Well-being and Aging in Latin America and the Caribbean (Salud, Bienestar y Envejecimiento en America Latina y el Caribe, or SABE) was a multi-centre cross-sectional survey of over 10 500 older adults (aged ≥ 60 years in 1999) conducted in the following seven cities in LAC between 1999 and 2000: Buenos Aires, Argentina; Bridgetown, Barbados; Sao Paulo, Brazil; Santiago, Chile; Havana, Cuba; Mexico City, Mexico; and Montevideo, Uruguay. The study, which received technical support from the Pan American Health Organisation (PAHO), comprised a research team which included members from PAHO, the University of Wisconsin-Madison, and local investigators from each collaborating city [9]. The survey collected comprehensive information on health, functional ability and social support networks. Ethical approval was granted for the conduct of the survey by the appropriate ethics review board in each city. Anonymised data from this study have been available to the public for free download since January 2005, from the National Archive of Computerized Data on Aging [10], and details about the methodology of the SABE study have already been published [9].

We analysed the SABE dataset for all seven cities, focusing on demographic data (age, sex, city), health status (self-reported health status, and presence of chronic disease or condition), functional status (activities of daily living, ADL), depression (Geriatric Depression Scale, GDS) and five levels of active community engagement.

### Health status

Participants had been asked whether their health was excellent, very good, good, fair or poor. We grouped responses into "good or better" self-reported health and "less than good". Participants had also been asked whether a doctor or nurse had ever told them that they had any of seven chronic diseases or conditions (diabetes, cancer, hypertension, heart problems, stroke, arthritis or chronic lung disease). We then created two summary disease indicators, which recorded whether a participant had reported having (a) at least one of these ('disease'); or (b) at least two of these (co-morbidity). We created a summary indicator for depression using the 15-item GDS [11], and used this when assessing the association of health status and functional status with active community engagement (see below).

### Functional status

We investigated limitations using six basic ADLs. These measure the independent performance of personal care tasks (dressing, eating, bathing, walking across a room, getting into/out of bed and using the toilet). They are useful for guiding clinical management decisions and care policies for the elderly [12], and are often used to measure lack of physical disability in the elderly. We created a summary "disability" indicator, defined as those who responded that they had had "difficulty in performing" at least one of the ADLs. This disability indicator was used to calculate disability-free life expectancy (LE) in years.

### Community engagement

We measured five levels of decreasing community engagement, from active through passive to very little involvement; as follows: (a) voluntary or paid employment; (b) active help for others (e.g. through providing some kind of service, like housework, transport, childcare); (c) passive help for others (providing money, clothes or food); (d) weekly community contact; or (e) less than weekly community contact.

### Statistical methods

To investigate the first aspect of active ageing (relating to lack of, or limited disability and disease), we calculated the prevalence of disability (ADLs) and of self-reported disease and co-morbidity, within each country and overall. We then calculated healthy LE following Sullivan's method [13], which uses life tables and prevalence data to estimate the number of remaining life years free from disability or disease. We obtained life tables online from the WHO [14]. We calculated adjusted LE estimates with and without (a) disability; (b) disease; and (c) co-morbidity; by sex and 5-year age-groups (60–64, 65–69, 70–74, 75–79, 80–84, 85–89 years, and 90 years and over), for each city and overall.

Next we constructed a log-linear regression model to examine the association of sex and location with disability-free LE (in years). We fitted three models. Firstly, for the location association, we compared each city to the regional average (adjusted for age). Secondly, for the sex association we compared men to women within each age group, over the whole region (we grouped the oldest three age-groups due to small numbers). Finally, for the sex association (adjusted for age and location) we compared sex within each city.

We also calculated the prevalence of each level of active engagement with community life. We constructed a second series of models to examine the association of the first aspect of active ageing (disease and disability) with the second aspect (active community engagement). Using our measure of disease as the only predictor, we fitted two ordinal-logistic regression models: one pre-adjusted for age, sex and location, and a second which also adjusted for depression. We then repeated this modeling process, replacing the disease measure with our co-morbidity and then our disability measure.

For participants missing 1, 2, or 3 GDS items, scores were scaled by number of unanswered items. We chose "3 items" as the maximum acceptable level of item non-response. For participants missing a single item, their scaled GDS score became: (Scaled GDS) = (original GDS) + (original GDS)*(15/14), and so on. This increased the proportion of participants with usable GDS scores from 73% to 98%. We performed a sensitivity analysis of our active engagement ordinal-logistic regression to assess the effect of this scaling on results. The scaled GDS produced more conservative regression estimates than either the original GDS or the unscaled GDS among participants answering 12 or more GDS items.

We weighted all prevalence estimates and individual-level regression analyses to account for the survey design and levels of non-response. We used Stata statistical software (Version 10, StataCorp LP, College Station, Texas, USA) for all analyses. We assumed statistical significance at p = 0.05, but present confidence intervals, and p-values where appropriate, to clarify the exact strength of statistical relationships.

## Results

### Prevalence of indicators by age, sex, and location

The SABE questionnaire response rates were 63% in Buenos Aires, 65% in Montevideo, 80% in Bridgetown, 84% in Santiago, 85% in Sao Paulo and Mexico City, and 95% in Havana [15] Table 1 details characteristics of the respondents in each of the seven capital cities. Overall, the mean age of participants was 70 years, with about 74% aged between 60 and 74 years; the majority (60%) were women.

**Table 1:** Age and sex distribution of adults aged 60 years and over in seven cities in Latin America and the Caribbean*

**Selected characteristics**	**City**							
	**Buenos Aires**	**Bridgetown**	**Sao Paulo**	**Santiago**	**Havana**	**Mexico City**	**Montevideo**	**All**
**Mean age (yr)**	70.7	72.4	69.4	70.3	71.1	69.7	70.9	70.3
**Age-group (yr)** ***N***	1043	1508	2143	1301	1905	1247	1450	10597
60–64 (%)	22.0	20.1	32.3	29.5	27.9	32.4	21.2	27.2
65–69 (%)	25.1	22.2	26.8	24.2	21.9	24.3	25.9	24.9
70–74 (%)	25.4	20.3	18.8	18.6	19.3	19.3	22.8	21.5
75–79 (%)	15.0	16.3	11.2	14.3	13.7	11.4	17.0	13.5
80–84 (%)	7.8	11.3	6.2	7.3	8.4	6.5	8.6	7.3
85–89 (%)	4.0	6.2	3.3	4.4	5.7	4.7	3.5	4.2
90 years & over (%)	0.8	3.5	1.4	1.7	3.1	1.5	1.0	1.3

**Sex: female *n*** **(%)**	660 (61.7)	924 (60.3)	1262 (58.6)	855 (59.8)	1197 (59.1)	740 (56.4)	920 (63.7)	**6558** **(59.7)**

*All participants provided information on age and sex.

The proportion of participants reporting good, very good or excellent health was 50% overall (ranging from 31% in Mexico City to 65% in Buenos Aires). Those reporting difficulties with at least one ADL ranged from 14% in Bridgetown to 22% in Santiago (the mean for the LAC region was 19%). In every city, over two-thirds of the elderly reported having at least one disease, ranging from 69% (Mexico City) to 82% (Buenos Aires); the regional mean was 77%. Co-morbidity was experienced by 44% of all participants (from 35% in Mexico City to 50% in Havana). The city with the greatest proportion affected by both disability and any disease, and both disability and co-morbidity, was Santiago (19% and 14%, respectively), while Mexico City had the highest proportion both disability-free and disease-free, at 28% (Table 2).

**Table 2:** Prevalence (%) of self-reported health status, disability, disease and co-morbidity in adults aged 60 years and over in seven cities in Latin America and the Caribbean

	Buenos Aires	Bridgetown	Sao Paulo	Santiago	Havana	Mexico City	Montevideo	All*
***N***	1043	1508	2143	1301	1905	1247	1450	10597
Self-reported health status^† ^% (95% CI)	65.2 (59.3–71.2)	51.8 (49.0–54.6)	47.7 (43.8–51.5)	40.0 (32.9–47.1)	37.3 (34.7–39.9)	31.2 (27.5–34.9)	61.4 (52.6–70.2)	**49.7** **(45.4–53.9)**
Neither disability nor disease % (95% CI)	17.0 (13.8–20.3)	21.9 (19.6–24.1)	20.7 (18.4–23.0)	18.4 (14.7–22.0)	17.7 (15.9–19.5)	27.8 (25.2–30.4)	20.6 (17.9–23.3)	**20.7** **(19.0–22.4)**
Any disability^‡ ^% (95% CI)	17.5 (14.6–20.4)	14.2(12.1–16.3)	19.2 (16.7–21.7)	22.1 (18.6–25.7)	19.1 (17.2–21.0)	19.3 (16.8–21.9)	17.3 (13.6–21.1)	**18.8** **(17.7–19.9)**
Any disease^‡ ^% (95% CI)	81.7 (78.6–84.7)	76.2 (73.8–78.6)	77.2 (74.9–79.5)	78.9 (74.8–83.0)	80.6 (78.7–82.4)	68.6 (66.0–71.1)	77.7 (75.5–80.0)	**77.1** **(75.3–78.9)**
Co-morbidity^§^% (95% CI)	46.2 (43.6–48.9)	44.7 (41.9–47.6)	46.1 (43.6–48.6)	45.0 (39.9–50.2)	50.2 (47.8–52.5)	34.7 (31.7–37.8)	46.9 (42.2–51.6)	**43.7** **(42.1–45.3)**
Disability and any disease^§^% (95% CI)	16.2 (13.0–19.4)	12.2(10.1–14.2)	17.1 (14.9–19.4)	19.3 (15.9–22.8)	17.3 (15.4–19.2)	15.6 (13.3–18.0)	15.8 (11.9–19.7)	**16.6** **(15.4–17.7)**
Disability and co-morbidity^§^% (95% CI)	11.8 (9.2–14.5)	8.3 (6.6–9.9)	12.6 (11.0–14.3)	14.1 (11.4–16.7)	12.1 (10.6–13.7)	10.5 (8.4–12.6)	11.7 (7.8–15.6)	**11.9** **(11.0–12.8)**

*The number of participants not providing information was: Any disability (*N *= 74; 0.7%); Any disease (*N *= 87; 0.8%); Co-morbidity (*N *= 167; 1.4%); Disability and any disease (*N *= 159; 1.4%): Disability and co-morbidity (*N *= 236; 2.0%); Neither disability nor disease (*N *= 159; 1.4%).

^†^"Self-reported health status" was defined as the proportion of participants reporting good, very good, or excellent health. Self-reported health status was not used if, due to cognitive limitations of the respondent, the question was answered by a substitute informant. Overall, 793 (6.2%) used a substitute informant and so could not provide valid self-reported health status.

^‡^"Any disability" was defined as difficulty in performing at least one of the six ADL activities (dressing, eating, bathing, walking across a room, getting into/out of bed and using the toilet). "Any disease" was defined as presence of at least one of the seven self-reported diseases or conditions (heart problems, cancer, stroke, hypertension, diabetes, arthritis, chronic lung disease; for prevalence of each individual disease see Appendix).

^§^'Co-morbidity" was defined as presence of at least two of the seven self-reported diseases or conditions. "Disability and any disease" was defined as difficulty in performing at least one of the six ADL activities and presence of at least one of the seven self-reported diseases or conditions. "Disability and co-morbidity" was defined as difficulty in performing at least one of the six ADL activities and presence of at least two of the seven self-reported diseases or conditions.

### Life expectancies with and without disability, disease and co-morbidity

**Figure 1:**
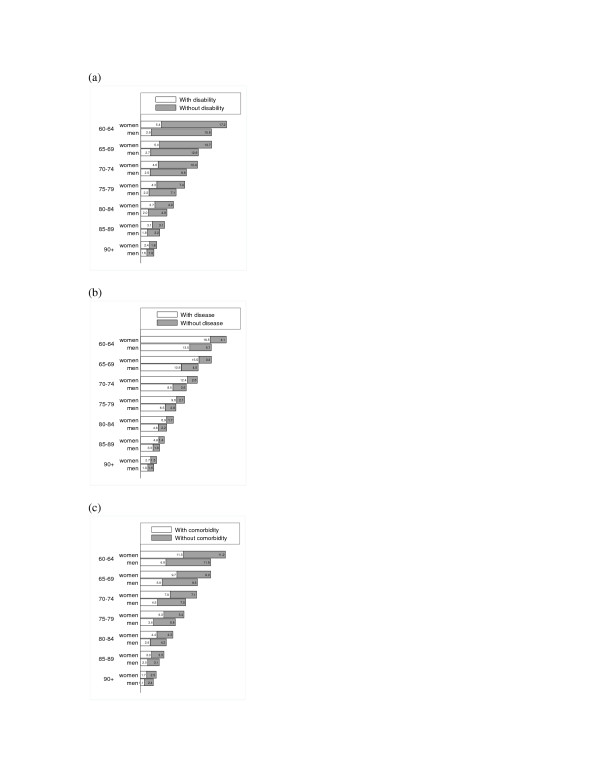
**1a – Life expectancy in years, with and without (a) disability^a^, (b) disease^b^, and (c) co-morbidity^c^,for men and women aged 60 years and over in seven cities in Latin America and the Caribbean.**^a^Disability was defined as difficulty with at least one of the six ADL activities (dressing, eating, bathing, walking across a room, getting into/out of bed and using the toilet). ^b^Disease was defined as presence of at least one, and ^c^Co-morbidity was defined as presence of at least two, of the seven self-reported chronic diseases or conditions (heart problems, cancer, stroke, hypertension, diabetes, arthritis, or chronic lung disease).

In Figures 1(a)–(c) we present the LE in years for elderly men and women in the LAC region, indicating years spent with and without disability, any disease and co-morbidity, respectively. In every age-group women had a longer LE than men; however, they also had more years with each condition. For example, women aged 60–64 years could expect 5 years to be spent with a disability (vs 3 years for men), 19 years with at least one chronic disease or condition (13 years for men), and 12 years with co-morbidity (vs 7 years for men).

**Figure 2:**
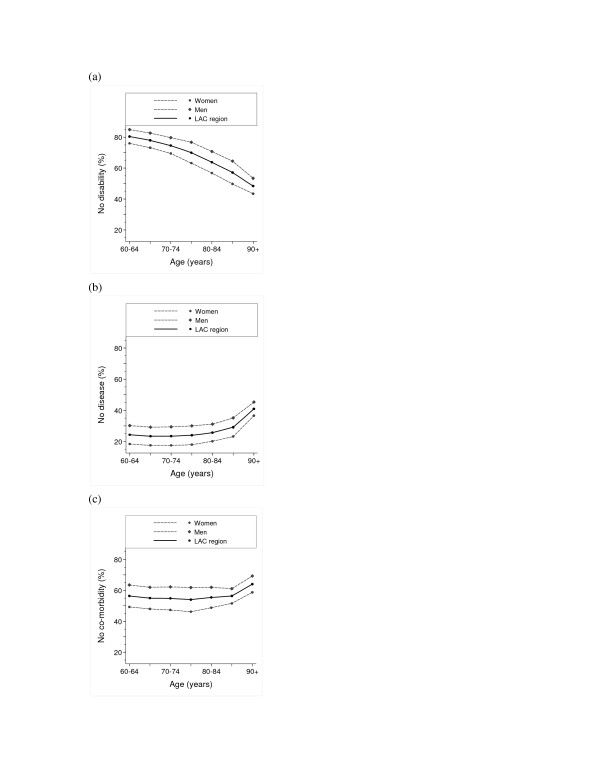
**2a – Proportion of remaining life expectancy without (a) disability^a^, (b) disease^b^, and (c) co-morbidity^c^,in men and women aged 60 years and over in seven cities in Latin America and the Caribbean. **^a^Disability was defined as difficulty with at least one of the six ADL activities (dressing, eating, bathing, walking across a room, getting into/out of bed and using the toilet). ^b^Disease was defined as presence of at least one, and ^c^Co-morbidity was defined as presence of at least two, of the seven self-reported chronic diseases or conditions (heart problems, cancer, stroke, hypertension, diabetes, arthritis, or chronic lung disease).

Figures 2(a)–(c) show the proportion of LE to be spent without disability, any disease and co-morbidity, respectively. For both sexes, (a) the proportion of disability-free LE declined with older age (from 85% to about 55% for men and from about 75% to about 45% for women); (b) disease-free LE remained at approximately 30% for men and 20% for women, until 80–84 years of age, then increased; and (c) co-morbidity-free LE remained at approximately 62% for men and 48% for women until 80–84 years of age, then increased.

Potential age and sex differences in LE were further examined in the regression model, as well as city differences. Compared with the youngest age-group (60–64 years), those aged 70 years and above had statistically significantly less disability-free LE (Table 3). For the entire LAC region, men had 8% more disability-free LE than women (incidence rate ratio, IRR = 1.08; 95%CI 1.06–1.11; p < 0.001) (Table 3). Only Bridgetown's participants had disability-free LE which was statistically significantly longer than the regional average (IRR = 1.08; 95%CI 1.05–1.10; p < 0.001), while only Santiago participants had disability-free LE which was shorter than the regional average (IRR = 0.94; 95%CI 0.92–0.97; p < 0.001) (Table 3). The sex differences in disability-free LE within each city were small (ranging from 5% longer disability-free LE for men than women in Mexico City, to 12% in Montevideo), and only in Mexico City was this difference not statistically significant.

**Table 3:** Disability-free life expectancy in adults aged 60 years and over in seven cities in Latin America and the Caribbean*

				**Sex^†^**		
		
	**IRR**	**95% CI**	**p-value**	**IRR**	**95% CI**	**p-value**
**Age (years)**						
60–64	1.00	-	-	1.06	1.02–1.10	0.003
65–69	0.98	0.95–1.01	0.14	1.06	1.02–1.11	0.002
70–74	0.96	0.93–0.98	0.003	1.03	0.98–1.08	0.23
75–79	0.87	0.85–0.90	< 0.001	1.13	1.07–1.19	< 0.001
80+	0.71	0.69–0.74	< 0.001	1.20	1.14–1.28	< 0.001

**City^‡^**						
Buenos Aires	1.01	0.98–1.04	0.48	1.08	1.01–1.15	0.02
Bridgetown	1.08	1.05–1.10	< 0.001	1.05	1.00–1.11	0.05
São Paulo	0.98	0.96–1.00	0.06	1.09	1.04–1.14	< 0.001
Santiago	0.94	0.92–0.97	< 0.001	1.09	1.03–1.16	0.004
Havana	1.00	0.98–1.02	0.86	1.09	1.04–1.14	0.001
Mexico City	0.98	0.96–1.01	0.20	1.05	0.99–1.11	0.14
Montevideo	1.01	0.99–1.04	0.24	1.12	1.06–1.19	< 0.001

*All participants provided information on age and sex.

^†^The sex association was pre-adjusted for age in the five age-groups, as well as for city. The unstratified effect of sex on disability-free life expectancy was 1.08 (95% CI 1.06 – 1.11) p < 0.001).

^‡^Each city was compared with the regional average (defined as the mean of all seven cities); pre-adjusted for age.

### Community engagement

Table 4 presents the prevalence of each level of community engagement measured, for elderly men and women. Fewer than 9% of women and 5% of men experienced less than weekly community contact (the lowest level of engagement). Seventy-four per cent of women and 77% of men were actively engaged in their community (the two highest levels of engagement: active help and voluntary or paid employment). In all cities, approximately one-third to one-half of all men maintained the highest level of active engagement (having paid or voluntary employment). In contrast, this level of engagement was maintained by between one-fifth and one-third of women in all cities except Havana, where fewer than one-tenth had paid or voluntary employment.

**Table 4:** Prevalence (%) of active engagement* with community life in women and men aged 60 years and over in seven cities in Latin America and the Caribbean

	**Buenos Aires**	**Bridgetown**	**Sao Paulo**	**Santiago**	**Havana**	**Mexico City**	**Montevideo**	**All**
***N***	1043	1508	2143	1301	1905	1247	1450	10597
**Women %** **(95%CI)**								
1) No regular community contact	11.4 (8.2–14.7)	15.5 (12.9–18.0)	4.5 (3.4–5.7)	3.9 (2.7–5.0)	12.1 (9.8–14.5)	9.9 (7.8–12.1)	7.3 (6.5–8.1)	8.9 (7.6–10.2)
2) Weekly community contact	10.0 (8.1-11.8)	10.6 ()8.6–12.6	7.1 (5.7–8.4)	9.4 (6.7–12.1)	12.9 (10.7–15.2)	13.6 (10.8–16.5)	7.1 (4.7–9.5)	10.2 (9.2–11.3)
3) Passive help	5.6 (4.3–5.1)	7.3 (5.3–9.3)	4.6 (3.5–8.4)	11.3 (8.5–14.2)	4.8 (3.5–6.1)	7.7 (5.6–9.8)	9.4 (7.7–11.1)	6.8 (5.8–7.8)
4) Active help	47.0 (43.0–51.0)	31.7 (27.9–35.4)	45.1 (41.7–48.5)	46.5 (41.7–51.3)	61.2 (57.8–64.7)	49.9 (45.6–54.2)	55.3 (50.3–60.4)	48.8 (46.9–50.8)
5) Voluntary or paid work	26.0 (22.4–29.6)	35.0 (31.3–39.0)	38.7 (35.9–41.6)	28.9 (24.0–33.9)	8.9 (7.0–10.8)	18.9 (16.0–21.7)	20.8 (14.0–27.6)	25.2 (23.5–26.9)
**Men %** **(95%CI)**								
1) No regular community contact	7.2 (4.1–10.3)	12.7 (9.8–15.6)	3.3 (2.0–4.6)	2.3 (0.9–3.7)	6.6 (4.7–8.6)	3.5 (1.9–5.0)	3.9 (1.9–5.9)	4.8 (3.8–5.9)
2) Weekly community contact	5.6 (3.7–7.6)	10.2 (7.4–13.0)	5.7 (3.4–8.0)	8.9 (6.0–11.8)	8.3 (6.3–10.4)	11.3 (8.5–14.0)	5.1 (3.0–7.2)	7.6 (6.6–8.8)
3) Passive help	7.0 (3.8–10.3)	13.4 (10.5–16.3)	10.9 (8.3–13.4)	15.0 (12.4–17.7)	6.1 (4.3–7.8)	12.1 (9.2–15.0)	17.8 (13.7–21.8)	10.6 (8.9–12.3)
4) Active help	35.4 (31.7–39.2)	25.2 (21.1–29.3)	25.7 (21.9–29.5)	26.4 (21.0–31.8)	43.9 (39.9–47.9)	25.0 (21.1–28.9)	45.8 (41.7–49.9)	31.5 (29.0–34.0)
5) Voluntary or paid work	44.7 (39.7–49.7)	38.5 (34.3–42.7)	54.4 (49.9–58.9)	47.4 (42.6–52.3)	35.1 (31.3–38.9)	48.2 (43.6–52.8)	27.4 (23.0–31.9)	45.5 (42.9–48.1)

*The 5 levels of community engagement were defined as follows:

(1) See or speak with offspring, siblings, other family, or friends less than once a week; (2) See or speak with offspring, siblings, other family, or friends at least once a week; (3) Giving money, or giving things (like food, clothing etc) to offspring, siblings, other family, or friends; ± Level (2); (4) Helping offspring, siblings, other family, or friends with services (such as transportation, housework etc), or childcare; ± Level (2) ± Level (3); (5) Provided services for free (to social services, children's home, senior citizen's centre, college or university, health centre, church, hospital, or other place), or worked for free or for payment in the past week; ± Level (2) ± Level (3) ± Level (4). Active engagement was then defined as a combination of Levels (4) and (5).

Community engagement decreased strongly with older age. Compared with those aged 60–64 years, older age-groups were less likely to report higher levels of participation in the community: for those aged 65–69 years, OR = 0.59 (95% CI 0.52–0.68); for the age-group 70–74 years, OR = 0.42 (0.36–0.50); for 75–79 years, OR = 0.28 (0.23–0.34); and for those aged 80 years and over, OR = 0.13 (0.11–0.16) (data not shown). A statistically significant sex difference was also found, with men more likely than women to be actively engaged in the community (OR = 1.89; 95%CI 1.66–2.15; p < 0.001) (data not shown).

After pre-adjusting for age, sex, and location, we show (Table 5) that the presence of disease and co-morbidity further limits community engagement in men only: those with disease or co-morbidity are about 30% less likely to have a higher level of engagement (disease: OR = 0.78, 95%CI 0.62–0.99, p = 0.04); co-morbidity: OR = 0.78, 95%CI 0.65–0.94, p = 0.01).

**Table 5:** Active engagement with community life among women and men aged 60 years and over in seven cities in Latin America and the Caribbean

	**Not adjusted for depression**			Adjusted for depression*		
**Condition**	**OR^†^**	**95% CI**	**p-value**	**OR^†^**	**95% CI**	**p-value**
		
**Women**						
Any disease^‡^	0.79	0.67–0.94	0.01	0.86	0.72–1.02	0.08
Co-morbidity	0.85	0.73–0.97	0.02	0.91	0.78–1.05	0.21
ADL^§^	0.52	0.44–0.61	<0.001	0.58	0.49–0.69	<0.001

**Men**						
Any disease^‡^	0.70	0.56–0.87	0.001	0.78	0.62–0.99	0.04
Co-morbidity^‡^	0.69	0.58–0.83	<0.001	0.78	0.65–0.94	0.01
ADL^§^	0.40	0.30–0.53	<0.001	0.50	0.38–0.67	<0.001

**All**						
Any disease^‡^	0.74	0.65–0.86	<0.001	0.82	0.71–0.94	0.01
Co-morbidity^‡^	0.79	0.71–0.87	<0.001	0.86	0.77–0.96	0.01
ADL^§^	0.48	0.41–0.56	<0.001	0.55	0.47–0.65	<0.001

*Models additionally adjusted for depression as a continuous variable, using the 15-point Geriatric Depression Scale. Note that depression alone (pre-adjusted for age, sex, and city) statistically significantly reduces active engagement: OR = 0.92 (95% CI 0.90–0.93); p < 0.001. For definition of active engagement see text (or footnote to Table 4).

^†^Models adjusted for age in the five age-groups (60–64, 65–69, 70–74, 75–79, 80+ years), by sex, and by city.

^‡ ^"Any disease" defined as presence of at least one of seven diseases or conditions (heart problems, cancer, stroke, hypertension, diabetes, arthritis, chronic lung disease; for prevalence of each individual disease see Appendix). "Co-morbidity" defined as at least two of the seven diseases/conditions.

Depression had a statistically significant association with community engagement, after adjusting for age, sex and city (OR = 0.92; 95% CI 0.90–0.93; p < 0.001) (Table 5). Further adjusting for depression weakened the association of summaries of disease and disability: for women, depression weakened the association of active engagement with disease by 11% (15% for men) and the association with co-morbidity by 8% (17% for men), although these were not statistically significant. However, the disability measure did remain statistically significant after adjusting for depression: being limited by 20% for women and 50% for men (p < 0.001 in each case) (Table 5). Although causality cannot be determined (does depression reduce community participation, or vice versa?), there was a clear association between disease or disability status, level of depression and community participation.

## Discussion

Our analyses showed that, overall, for every 100 persons over the age of 60 years in LAC, 77 had at least one disease, 44 had at least two diseases, 19 had a disability, 17 had both disability and at least one disease, 12 had disability and at least two diseases, and 21 had neither disability nor disease. Women, who had longer LE than men, could expect to have more of these remaining years spent with disability. The proportion of remaining disability-free years declined with older age for both men and women. Statistically significant differences in both sex and location were observed in the remaining disability-free years, after adjusting for age. The burden of disease was high (77%) and remained so up to the age of 85 years. Despite this, about half of all participants reported their health status as good or better. The city with the lowest self-reported good (or better) health status (Mexico City, at 31%) was also the city with the greatest proportion of participants without either disability or any disease (28%). Approximately three-quarters of the elderly LAC population were actively engaged in their communities. Although overall prevalence of active engagement was similar for men (77%) and women (74%), a greater proportion of men were involved in voluntary or paid work (46% vs 25%), while more women were actively helping others in the community (49% vs 32%). Active engagement diminished with increasing age and was limited by disability, any disease and co-morbidity. These associations were weakened by depression, although only for men did all associations remain statistically significant after adjustment for depression (for women, only disability retained statistical significance).

Our results confirm those of others which have shown that, despite their longer expected life, women could expect to spend more of it with reduced functional capacity than men [16-20]. Although not much is known about disability rates in LAC countries at the national or regional level, some preliminary data on ADL levels in the region have been published [21], and similar results to ours were demonstrated for Brazil, using the SABE dataset [22].

Other studies on LAC elderly, while not considering LE, have investigated disability [23] or disease, finding sex differences in functional impairment [24,25], and association of disability with some chronic diseases [26-28]. Few of these studies, however, considered all countries involved in the SABE study [23,26] and ours is the only analysis of disability-free LE that includes the whole LAC region. We show similar findings (more social networking associated with less disability) to those of longitudinal studies investigating social networking and disability in the elderly in different communities [29-32] However, to our knowledge, this paper is the first to estimate community engagement as part of a definition of active ageing in the LAC region.

All analyses in this study have been weighted to adjust for sampling design and for the level of survey non-response. However, while these weights adjust for *overall *level of response (questionnaire non-response), they are not designed to adjust for differential response rates to specific questions (item non-response). While the SABE data contain very high (over 99%) response rates from both males and females to questions regarding ADL activities, different groups of participants (such as men and women, or the elderly in different cities) may have different ways of responding, related to their level of disability. This concept is known as differential item functioning [33]. Further work is needed to assess the impact of differential item functioning on disability reporting in the elderly.

Our analyses could not show which particular diseases (perhaps those that would limit ADL) might have been driving the association with active community engagement, but it is likely that some (e.g. hypertension) may have less of a disabling association than others (e.g. arthritis). Depression increases the risk of functional disability in the elderly, due to decreased physical activity and less community engagement [34,35]. However, depression might also occur as a result of disease-related disability. It is also possible that this condition plays a part in modifying survey responses.

Our analyses had several limitations. Firstly, the data we used were from a cross-sectional study which was not powered to investigate interactions between variables. In addition, there could have been selection bias from non-response to the survey, which was below 17% for all but two of the cities, or from item non-response, although in general this was very low: below 2% for all variables analysed except self-reported health status (6%) and depression, which was adjusted by scaling for non-response; discussed in detail in the Methods. A sensitivity analysis (to assess the effect of this scaling on our results) led us to conclude that our GDS scaling was able to increase numbers used on the model (and so offer increased precision) without adversely affecting the point estimates.

In addition, to calculate disability-free, disease-free and co-morbidity-free LE, we used WHO life tables. These are created from WHO's World Health Report data, using country-wide population estimates, while the data used in our analyses were taken from the elderly population in large urban centres (i.e. not including rural areas). Depending on the proportion of rural to urban elderly, and on whether rural elderly populations have longer or shorter disease-free (and other) LE than those in cities, this could have either resulted in an over- or underestimation of LE projections. Finally, our measure of community engagement has not been validated.

We used the Rowe and Kahn model together with the WHO definition as a framework to estimate aspects of active ageing in the elderly LAC community. Some overlap exists between Rowe and Kahn's three elements: both physical and cognitive function are needed for active engagement in the community, while absence of disability implies presence of physical ability. Hence we chose not to assess physical and cognitive function, but instead focused on the other two elements (disease/disability and active community participation).

The challenge for public and private institutions in meeting the demands of older populations will depend to a large extent on the levels of co-morbidity or disability experienced by older individuals in various communities, and the degree to which institutions are prepared for providing for the specific needs of their senior citizens. One important step in making these preparations is to understand the degree of co-morbidity and disability among the older community, and how they impact on LE.

## Conclusion

High levels of active engagement in the community were observed, despite prevalence of 77% for any disease/condition and 19% for disability, and their associated limitation of active engagement. Our results thus reinforce the importance of considering 'less disease' in the elderly rather than none, when assessing levels of active ageing. In addition, perhaps depression should also play a part in definitions of 'active ageing', due to its association with both disease and disability.

Estimates of disability-free LE and active community participation provide important determinants of the degree of social and economic support needed to meet future demand from this vulnerable group. This is invaluable information for nations in the LAC region as they begin to deal with projected increases in the elderly population. As these nations cope with the social and economic demands of an increasing burden of disease and disability in older age, opportunities should be maximized for community participation by this increasingly frail, but actively engaged group.

## Competing interests

The author(s) declares that they have no competing interests.

## Authors' contributions

AH and IRH conceived and designed the study and acquired the data. AMCR and IRH analysed and interpreted the data. AMCR drafted the manuscript. All authors critically revised the manuscript for important intellectual content. AH supervised the study. All authors saw and approved the final version of the manuscript.

## Appendix

See Table 6.

**Table 6:** Appendix. Prevalence of seven chronic non-communicable diseases or conditions in adults aged 60 years and over in seven cities in Latin America and the Caribbean*

	**City**							
Selected diseases/conditions (%)	**Buenos Aires**	**Bridge-town**	**Sao Paulo**	**Santiago**	**Havana**	**Mexico City**	**Montevideo**	**All**
***N***	1043	1508	2143	1301	1905	1247	1450	10597
	
Heart problems	19.8 (17.7–22.0)	11.1 (9.4–12.7)	19.6 (17.6–21.6)	32.2 (26.2–38.3)	24.1 (22.0–26.2)	10.0 (8.2–11.8)	23.8 (20.1–27.5)	**19.3** **(18.0–20.7)**
Stroke	4.8 (2.6–7.1)	5.4 (4.1–6.6)	7.2 (6.1–8.4)	6.5 (4.8–8.3)	9.7 (8.2–11.2)	5.8 (4.4–7.3)	3.7 (2.6–4.8)	**5.9 (5.0–6.8)**
Hypertension	49.7 (46.2–53.1)	47.5 (44.6–50.3)	53.7 (50.9–56.5)	51.4 (46.5–56.3)	44.1 (41.5–46.7)	42.8 (39.9–45.7)	45.2 (41.8–48.7)	**48.1 (46.6–49.6)**
Cancer	5.3 (4.0–6.5)	2.9 (2.0–3.8)	3.3 (2.5–4.1)	4.1 (3.0–5.3)	3.4 (2.6–4.2)	1.9 (1.1–2.6)	6.3 (5.0–7.5)	**4.0 (3.4–4.5)**
Diabetes	12.4 (8.5–16.2)	21.7 (19.3–24.1)	18.0 (15.9–20.1)	13.3 (10.0–16.5)	14.8 (13.0–16.6)	21.6 (19.0–24.2)	13.7 (10.9–16.5)	**15.9 (14.5–17.3)**
Arthritis	52.6 (49.3–56.0)	46.2 (43.2–49.1)	32.2 (29.9–34.6)	29.3 (26.3–32.3)	55.8 (53.5–58.1)	24.6 (21.8–27.3)	48.2 (44.5–51.9)	**40.1 (36.2–44.0)**
Chronic lung disease	7.8 (6.2–9.5)	4.1 (3.0–5.3)	12.2 (10.8–13.6)	11.9 (9.2–14.5)	12.9 (11.3–14.5)	9.8 (8.1–11.5)	9.3 (8.9–9.7)	**9.9 (9.0–10.7)**

*The number of participants not providing information was: heart problems (42; 0.3%), stroke (30; 0.2%), hypertension (52; 0.5%), cancer (35; 0.3%), diabetes (57; 0.5%), arthritis (71; 0.6%), chronic lung disease (28; 0.2%).

## Pre-publication history

The pre-publication history for this paper can be accessed here:


